# The Transmission of Animal African Trypanosomiasis in Two Districts in the Forest Zone of Ghana

**DOI:** 10.4269/ajtmh.23-0329

**Published:** 2024-05-02

**Authors:** Austine Tweneboah, Jana Rosenau, Kofi Agyapong Addo, Thomas Kwame Addison, Mahamat Alhadj Moussa Ibrahim, Judith Sophie Weber, Soerge Kelm, Kingsley Badu

**Affiliations:** ^1^ Department of Theoretical and Applied Biology, Kwame Nkrumah University of Science and Technology, Kumasi, Ghana;; ^2^ Department of Biology and Chemistry, University of Bremen, Bremen, Germany;; ^3^ Department of Biology, University of N’Djamena, N’Djamena, Chad

## Abstract

Animal African trypanosomiasis, also known as nagana, is caused by *Trypanosoma* species, which cause significant clinical diseases and lead to losses in animal production. We carried out a cross-sectional survey to investigate the composition of vectors and parasite diversity in two districts in the eastern region of Ghana where pigs and cattle were exposed to tsetse bites. We performed *cytochrome c oxidase subunit 1* polymerase chain reaction (PCR) to identify tsetse species and *internal transcribed spacer 1* PCR to identify *Trypanosoma* species. Also, we investigated the source of tsetse blood meal based on mitochondrial *cytochrome b* gene sequence analysis. A total of 229 tsetse, 65 pigs, and 20 cattle were investigated for trypanosomes. An overall vector density of 4.3 tsetse/trap/day was observed. A trypanosome prevalence of 58.9% (95% CI = 52.5–65.1%), 46.2% (95% CI = 34.6–58.1%), and 0.0% (95% CI = 0.0–16.1%) in tsetse, pigs, and cattle, respectively, was detected. *Trypanosoma congolense* was predominant, with a prevalence of 33.3% (95% CI = 73.3–86.5%) in tsetse. There was evidence of multiple infections in tsetse and pigs. Approximately 39% of the tsetse were positive for multiple infections of *T. congolense* and *Trypanosoma simiae*. Parasite prevalence in pigs across the communities was high, with significant differences associated between locations (χ^2^ = 28.06, 95% CI = 0.05–0.81, *P* = 0.0009). Tsetse blood meal analysis revealed feeding on domestic *Sus scrofa domesticus* (pigs) and *Phacochoerus africanus* (warthogs). Infective tsetse may transmit trypanosomes to livestock and humans in the communities studied.

## INTRODUCTION

Trypanosomiasis is a vector-borne infectious disease of major public health and economic importance in sub-Saharan Africa.^1^^–^^3^ It is a parasitic disease caused by flagellate protozoans belonging to the genus *Trypanosoma*. The parasites circulate in their host’s blood and central nervous system (humans and livestock) and are transmitted biologically by blood-sucking dipterans of the genus *Glossina*.^4^ The parasites can also be transmitted mechanically by other biting flies, including the tabanids.^5^ Several *Trypanosoma* species cause infection in humans and animals. *Trypanosoma brucei gambiense* determines the human chronic disease form in west and central Africa, whereas *Trypanosoma brucei rhodesiense* circulating in east and southern Africa transmits the acute form of the disease.^6^ The WHO estimates that 65 million people are at risk of trypanosomiasis, with a high proportion going without any medical monitoring.^7^ The disease in animals is known as the animal African trypanosomiasis (AAT), otherwise known as “nagana,” a word derived from Zulu that means powerless or useless.^8^ The parasite species that infect animals include *Trypanosoma congolense*, *Trypanosoma vivax*, *Trypanosoma brucei*, *Trypanosoma simiae*, and several other subspecies.^9^ Unlike in domestic animals, where severe infection usually leads to untreated death, nagana has a relatively mild effect on wild animals.^10^ At least 17,500 new cases are reported every year in livestock production.^11^

Animal African trypanosomiasis is mostly restricted to rural forested communities that rely heavily on agriculture.^6^ Approximately 15 million cattle are prone to infection in sub-Saharan Africa.^12^ The African continent is known to account for the high prevalence of animal trypanosomiasis, especially in tsetse-populated zones.^13^ However, information on the current situation of trypanosomiasis in Ghana remains scanty^14^ because AAT is generally neglected even at the national level in Ghana, and a tsetse control program is lacking.

Nagana causes huge economic losses in agriculture as a result of several factors, including reduced livestock production and the cost of treatment of infected livestock with poisonous drugs (e.g., Suramin).^15^ A wide range of animals serve as potential reservoirs of both animal and human forms of the disease, preventing significant livestock production over a large portion of the Saharan region. The major vector, *Glossina palpalis*, is widely distributed in Africa, and Ghana is no exception.^16^ In 2007, there was an outbreak of AAT that resulted in more than 50% losses of domestic pigs in the eastern region of Ghana.^17^ Subsequently, a few studies were carried out to identify the prevalence of animal pathogenic trypanosomes in both tsetse and livestock. The most recent investigation was done to assess the spatial distribution of tsetse in three districts, including the area where our study was conducted. That study revealed the presence of tsetse with *G. palpalis* in all three districts.^18^ *Trypanosoma brucei rhodesiense* is zoonotic,^19^ and that makes it difficult to control or obtain zero cases in humans because of the close relationship between humans and livestock. Thus, elimination should undoubtedly be geared toward vector control. The presence of tsetse is a public health challenge and a threat to livestock production.^20^ In the Suhum municipality, where our study took place, it is common knowledge that AAT killed a large number of infected pigs during the outbreak (H. Yao Gbeve, personal communication). Therefore, our study was carried out to determine the prevalence of *Trypanosoma* parasites in pig and cattle blood as well as the invertebrate host (vector) using molecular techniques to identify disease risk areas in the Suhum municipality for future studies

## MATERIALS AND METHODS

### Study area.

This study was conducted in selected communities in the Ayensuano and Kraboa districts (latitude 6°23.84′N, longitude 0°278.64′W) in the eastern region of Ghana. The region is positioned in the semideciduous forest zone and has an annual rainfall of 1,500 mm. Thus, most of the communities on the outskirts of the Suhum municipality have more than half of their population engaged in agriculture. The type of soil in the region is conducive to the cultivation of cash and food crops. As a result, crop farming and livestock rearing are the main agricultural activities in the district. Farmers in remote areas have satellite homes with small domestic farming as their supporting income or the main economic livelihood for the family. Two different areas within the municipality were visited in February 2018 and February 2019. Previous studies indicated no variation in transmission across climatic seasons.^21^

### Study design.

A cross-sectional community-based survey was used, in which pigs and cattle were sampled during the study period.

### Fly trapping and dissection.

**Figure 1.**
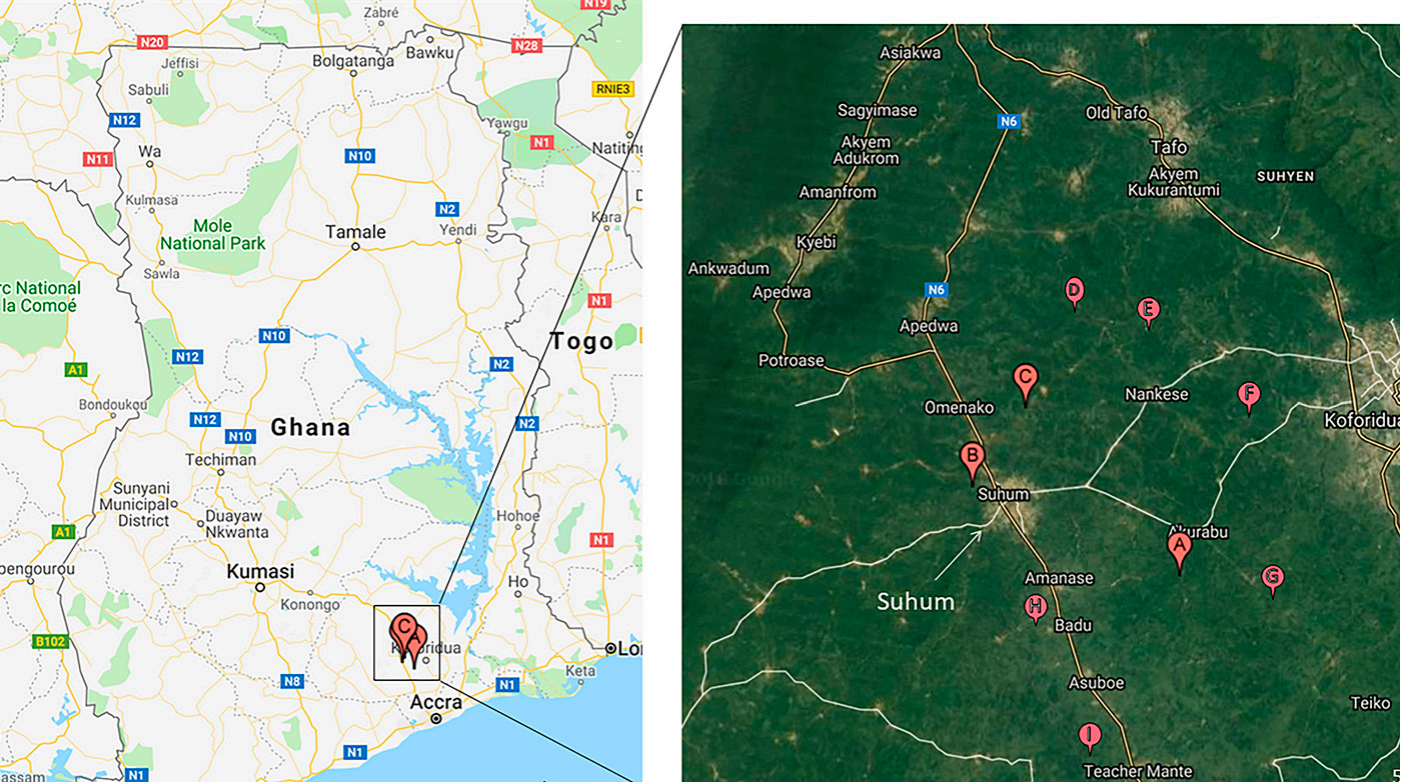
Tsetse were collected in some select communities in the Suhum, Kraboa, and Coaltar districts. Two traps were set up at Santramor (**A**), two at Pinto (**B**), two at Adenta (**C**), and one each at Nkatekwan (**D**), Zorh (**E**), Ayekokooso (**F**), Whanabenya (**G**), Magazine (**H**), and Teacher Mante (**I**).

**Figure 2.**
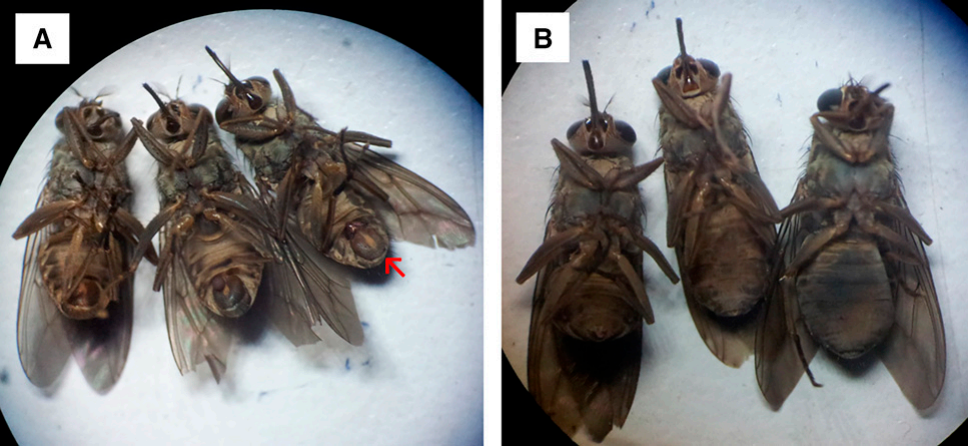
(**A**) Male tsetse trapped in the field were identified by the presence of a hypopygium (at the external genitalia, as indicated by the red arrow). (**B**) Female tsetse were identified by the absence of a hypopygium on the last segment of the abdomen.

We used unbaited biconical traps^22^^–^^24^ to collect tsetse from 13 communities. Two traps each were set up at Santramor, Zorh, Pinto, and Adenta and one each at Nkatekwan, Magazine, Teacher Mante, Bonkua No.1, Budu, Nankese, Mensakrom, Akorabo, Whanabenya, and Ayekokooso (Figure 1). Each trap was mounted close to the pigsties (∼5–10 m), but under a shade to minimize fly mortality. The coordinates of each trap were recorded with a Global Positioning System. After fly collection, an index of the apparent abundance of tsetse was calculated as the number of tsetse found per trap per trapping day.^25^ Tsetse were grouped into males or females based on the presence or absence of the hypopygium (a round structure on the last segment of the abdomen; Figure 2).^26^ Each fly was immobilized at 4°C before dissection, and the wings, legs, proboscis, and gut were each placed into 2-mL microcentrifuge tubes for preservation. However, only the guts and the proboscises were investigated for trypanosomes in our study.

### Blood sample collection.

We selected 65 pigs from all farms, including individual pigs from homes. The 20 cattle available in the community were also sampled. Six milliliters of blood was obtained from each cow from the jugular vein and was placed into a heparinized vacutainer. Approximately 3–4 mL of blood was drawn from the marginal ear vein of each pig into an ethylenediaminetetraacetic acid (EDTA) tube. After sampling, the vacutainers were kept in a refrigerator until further processing.

### DNA extraction.

#### Tsetse

The guts of the tsetse were homogenized in 50 mM Tris buffer (pH, 9.0) using a motor-driven homogenizer and micropestles (Kimble^®^ Kontes disposable pellet pestles; Sigma-Aldrich, St. Louis, MO), and 100 *µ*L of each homogenate was transferred into 1.5-mL Eppendorf tubes (Oldenburg, Germany). DNA was extracted from the gut using the DNeasy Blood and Tissue Kit (Qiagen, Hilden, Germany) according to the manufacturer’s instructions, and 60 *µ*L of each was eluted and stored at −20°C. DNA was isolated from the proboscises of the tsetse according to the manufacturer’s supplementary protocol on purification of total DNA from insects using the DNeasy Blood and Tissue Kit. Sixty microliters of the purified DNA was eluted and stored at −20°C until further processing.

#### Cattle and pig blood.

DNA was extracted from pig and cattle whole blood using the DNeasy Blood and Tissue Kit under the manufacturer’s protocol. The extracted DNA was quantified using a Nanodrop^®^ ND-1000 spectrophotometer (Thermo Fisher Scientific, Waltham, MA) at 260 nm.

### Polymerase chain reaction amplification of the *internal transcribed spacer 1* gene for the identification of *Trypanosoma* species.

The *internal transcribed spacer 1* (*ITS1*) region was amplified by nested polymerase chain reaction (PCR) to identify trypanosomes in tsetse gut, cattle, and pigs using the primers^27^ listed in Table 1. Each PCR was carried out in a total volume of a 25-*µ*L mixture containing 2 *µ*M of each primer, 0.2 mM of deoxynucleotide triphosphate mix, 1× buffer (DreamTaq), 0.1 U Taq polymerase, and molecular-grade water (all reagents from Thermo Fisher Scientific). The cycling conditions were the same for both nested reactions, with denaturation at 94°C for 1 minute, annealing at 54°C for 30 seconds, and extension at 72°C for 30 seconds. The reaction mixture was cycled 30 times for each PCR. The mixture was incubated at 95°C for 3 minutes before cycling, and there was a final extension at 72°C for 5 minutes after cycling. All PCRs were carried out in a Mastercycler Personal (Eppendorf, Hamburg, Germany). The resulting PCR products were electrophoresed on a 1.5% agarose gel run in Tris-borate–EDTA buffer at 100 V for 1 hour.^28^

**Table 1:** Primers, annealing temperatures, and targets used in our study

Primer	Direction of the Sequence (5′ → 3′)	Target	Annealing Temperature (°C)	Expected Band Size
*ITS* OutF *ITS* OutR *ITS*1 InF *ITS*1 InR	TGC AAT TAT TGG TCG CGC CTT TGC GTT CTT TAG AGG AAG CAA AAG AAG CCA AGT CAT CCA TCG	All *Trypanosoma* spp.	54	Variable
Tc*ITS*1-OutF Tc*ITS*1-OutR Tc*ITS*1-InF Tc*ITS*1-InR	TGCAATTATTGGTCGCGC TGTTGGTCGACACTGAGA TCGCGTGTCTCACGT TCAAAGATTGGGCAATGT	*Trypanosoma congolense*	54/56	681 bp (kilifi), 781 bp (forest)
*COI*-F *COI*-R	TTGATTTTTTGGTCATCCAGAAGT TGAAGCTTAAATTCATTGCACTAATC	Generic *Glossina*	55	900 bp
VF1d_t1 VR1d_t1	TGTAAAACGACGGCCAGTTCTCAACC- AACCACAARGAYATYGG CAGGAAACAGCTATGACTAGACTTCT- GGGTGGCCRAARAAYCA	All Vertebrates	55	500 bp

*COI* = *cytochrome c oxidase 1*; F = forward; In = inner primer; *ITS* = *internal transcribed spacer*; Out = outer primer; R = reverse; Tc = *Trypanosoma congolense*; VF1d_t1 =universal forward primer; VR1d_t1 = universal reversal primer.

### *Trypanosoma congolense*–specific PCR.

Samples that showed a band size of ∼700 bp with generic primers were further run with *T. congolense–*specific primers to confirm the presence of *T. congolense.* The species-specific primers used in a nested PCR also targeted the *ITS1* region of the parasite’s DNA. Sample preparation and PCRs were identical to *ITS1*-nested PCRs as described in the previous paragraph.

### Amplification of the *cytochrome c oxidase subunit 1* gene for the identification of tsetse species.

To identify the tsetse species in the study area, a part of the *cytochrome c oxidase subunit 1* (*COI*) gene^29^ was amplified in a one-step PCR according to the protocol described.^30^

### Blood meal analysis.

The extracted DNA from the tsetse gut was screened for the source of the tsetse blood meal in a one-step conventional PCR. One universal reverse primer and five vertebrate-specific (human, cow, goat, pig, and dog) forward primers were used for amplification of the mitochondrial *cytochrome b* gene to test for the specific host blood meal origin using conventional PCR.^31^^,^^32^

### Sequencing of *COI* fragments.

*Cytochrome c oxidase subunit 1* fragments were sequenced for the identification of tsetse species in the study area. The resulting gel product from *cox1* PCR was purified using the GeneJET Gel Extraction Kit (catalog no. K0701, Thermo Fisher Scientific) according to the manufacturer’s protocol. The gel containing the DNA fragments was excised with a clean scalpel into preweighed 1.5-mL Eppendorf tubes. The cut gels were purified according to the manufacturer’s protocol, and 50 *µ*L of each sample was collected in the elution buffer. DNA was quantified with a Nanodrop ND-1000 spectrophotometer at 260 nm. Twelve microliters of each amplicon containing 18 ng/100 bp was prepared for sequencing at Microsynth Seqlab GmbH (Göttingen, Germany). The sequences obtained were evaluated using Geneious v. 5.5.9.0 (Auckland, New Zealand). For purposes of comparison, the sequences were compared with the Nucleotide Blast database at the National Center for Biotechnology Information (NCBI)^33^ and the Tritryp database.^34^

## STATISTICAL ANALYSES

We processed our data using Excel (Microsoft Corporation, Redmund, WA) and transported it to GraphPad Prism for statistical analysis. Trypanosome distribution and infection rates in tsetse and pigs within communities were compared using a χ^2^ test, or Fisher’s exact test if an expected frequency was less than five. Differences in infection rates by sex of tsetse, by sex and age of pigs, and by community in both were checked. The comparisons were considered significantly different at a *P*-value <0.05.

## RESULTS

The average apparent density of tsetse during the survey period was 4.3 per trap per day. We collected 245 tsetse using an unbaited biconical trap^35^ and dissected 229 of them: 74 males and 155 females. Among the communities we visited, tsetse infestation was low at Whanabenya, whereas Adenta recorded the greatest infestation.

### Molecular identification of the tsetse.

Part of the *COI* gene of all the tsetse we collected during the survey was amplified and sequenced for species identification. Each sequence Basic Local Alignment Search Tool (BLAST) was performed and analyzed at both the NCBI and the Tritryp database, revealing a 100% similarity index with *G. palpalis* entries in the database. Subsequently, sequence analysis utilizing the neighbor-joining method resulted in the clustering of all tsetse as a single species, identified as *G. palpalis*.

### Molecular detection and identification of trypanosomes in the tsetse gut and proboscis.

We performed nested PCR with DNA extracted from tsetse gut tissue and the proboscis using generic primers to amplify the *ITS1* (5.8S and 18S) genes of Trypanosomatidae. Of all tsetse examined, 58.9% were positive for trypanosome DNA. The most common species found in the gut samples was *T. congolense* (33.3%), followed by *T. simiae* (14.1%). Some tsetse had multiple trypanosome parasites in their gut. The most frequent multiple infection was *T. congolense* and *T. simiae* (38.5%), followed by *T. congolense* and *T. brucei* species (7.4%) (Table 2). The proboscis had a relatively lower frequency of trypanosome DNA (16 of 200) compared with the gut (176 of 299). Overall, 16 of 200 proboscises were positive for the trypanosome *ITS1* sequence. *Trypanosoma congolense* was again the most commonly occurring species, with a prevalence of 6.5%. *Trypanosoma vivax* was also observed in the proboscis, with a frequency of 1.5%.^36^ The collection and density of tsetse reflected environmental conditions, including vegetation and the presence of livestock at the various sites. The largest number of tsetse was collected in the villages within the Suhum Kraboa District, where there was dense vegetation cover and unprotected pigsties. The distribution of *Trypanosoma* species varied among the communities, with a significant association among them (χ^2^ = 31.7, 95% CI = 34.6–58.1, *P* = 0.0002).

**Table 2:** The rate of occurrence of *Trypanosoma* species in tsetse and pigs collected in the field

*Trypanosoma* Species	Frequency (%)		
	Gut	Proboscis	Pigs
*Trypanosoma congolense/ Trypanosoma simiae*	38.5	0.0	3.3
*T. simiae*	14.1	0.0	20.0
*T. congolense*	33.3	6.5	60.0
*T. congolense/Trypanosoma vivax*	0.7	0.0	0.0
*T. congolense/Trypanosoma brucei* sp.	7.4	0.0	0.0
*T. brucei* sp.	3.7	0.0	0.0
*T. vivax*	1.5	1.5	16.0

**Figure 3.**
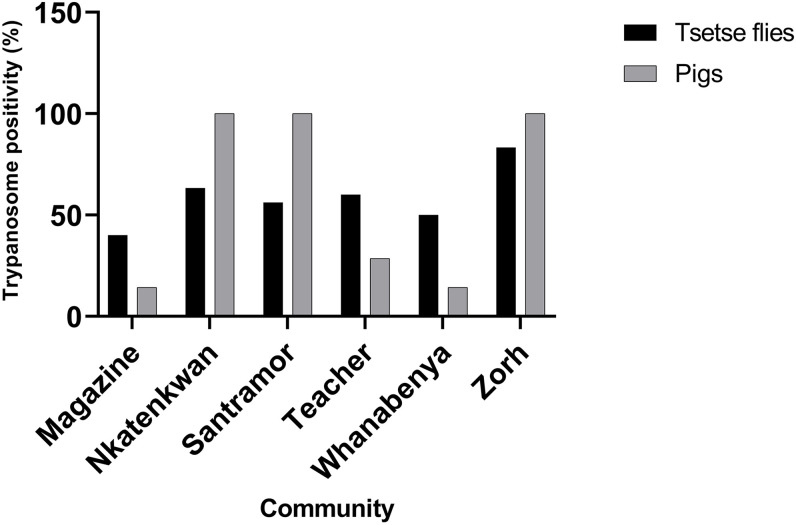
Relationship of trypanosome positivity between tsetse and pigs collected from the same communities.

The tsetse we collected in the field were observed to carry different trypanosome parasites in the gut and proboscis, and different trypanosomes were identified in the blood samples from pigs. The frequency of trypanosome DNA in tsetse was proportional to an increased rate of infection in pigs at the same collection site (Figure 3).

### *Trypanosoma congolense*–specific nested PCR.

**Figure 4.**
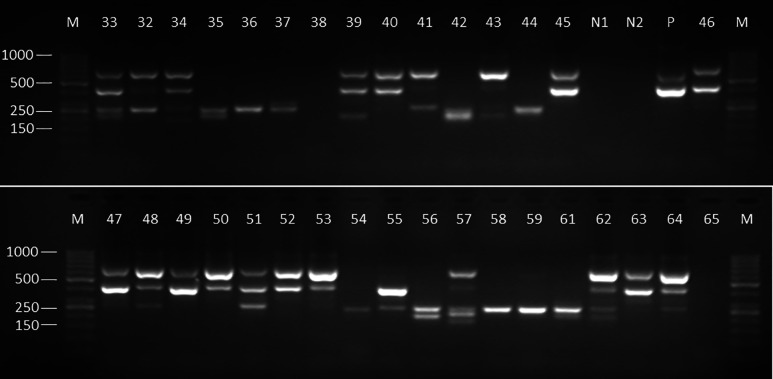
Gram-stained 2% agarose gel showing amplification of polymerase chain reaction products from tsetse gut infected with trypanosomes. Amplicons with sizes of ∼700 bp indicate *Trypanosoma congolense*, 420–500 bp derive from *Trypanosoma brucei*–like species (defined by their similar *internal transcribed spacer 1* length in our study), ∼370–400 bp correspond to *Trypanosoma simiae*, and ∼200–320 bp indicate *Trypanosoma vivax*. M = molecular marker GeneRuler 50-bp Ladder (Thermo Fisher Scientific); N1 and N2 = negative controls from both the first and second reactions; P = positive control (genomic DNA of *T. brucei*).

All tsetse and pig samples that were considered PCR positive for *T. congolense* were further subjected to species-specific nested PCR to confirm the presence of *T. congolense* and to determine the specific subspecies of *T. congolense* responsible for the infection. Of the 48 samples that were positive for *T. congolense* based on generic *ITS1* PCR, 46 of them had a band size of 760 bp (Figure 4), indicating the presence of forest type, and two samples did not show any band.

### Molecular detection and identification of trypanosomes in cattle and pigs.

**Figure 5.**
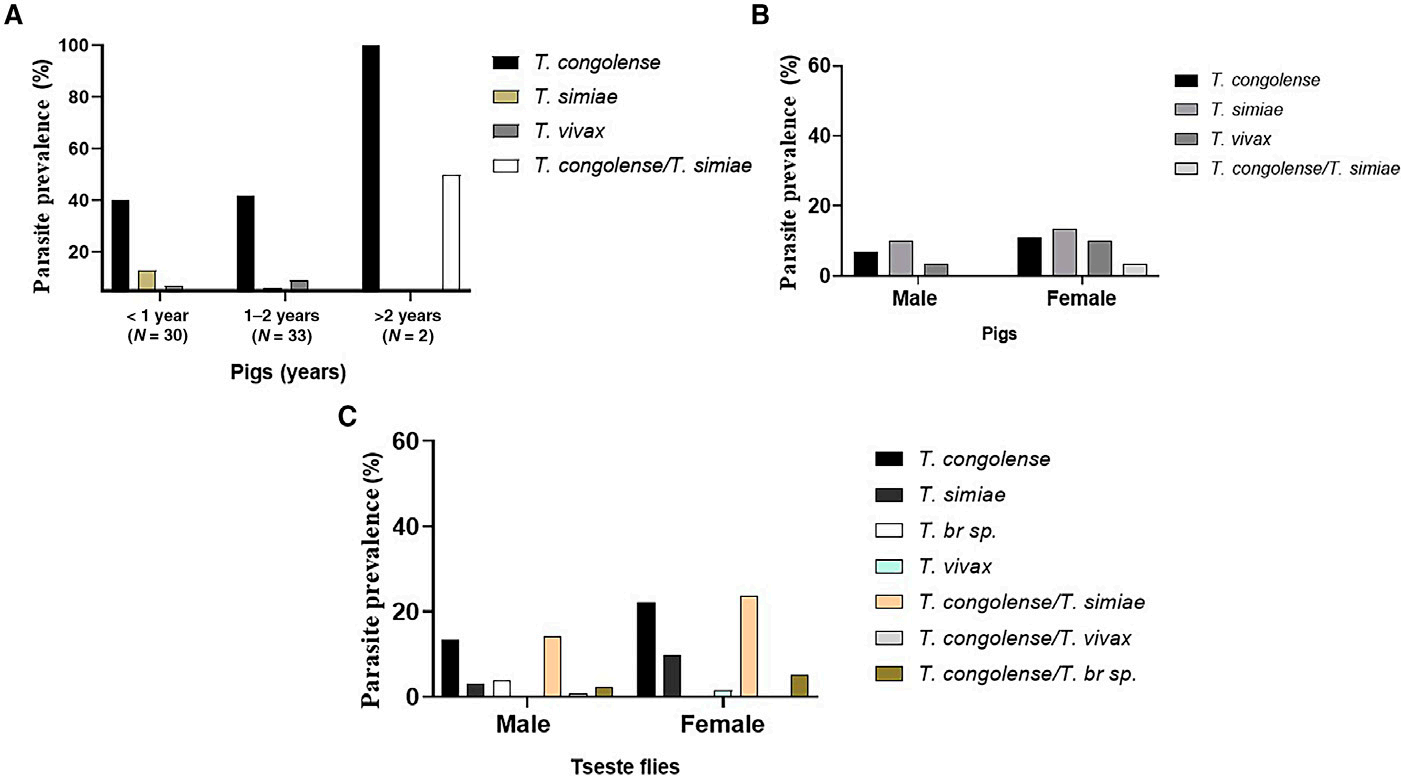
The relative distribution of single and multiple trypanosome DNA in tsetse and pigs from the eastern region of Ghana. All trypanosomes were identified by a part of the *internal transcribed spacer 1* gene. (**A**) Frequency of trypanosome in pigs in relation to age. (**B**) Frequency of trypanosome DNA in pigs (whole blood) in relation to sex. (**C**) Frequency of trypanosome DNA in tsetse in relation to sex. *T. br. sp = Trypanosoma brucei-like species*; *T. congolense* = *Trypanosoma congolense*; *T. simiae* = *Trypanosoma simiae*; *T. vivax* = *Trypanosoma vivax*.

Twenty cattle and 65 pigs from the selected communities were screened for the presence of trypanosomes by nested PCR targeting *ITS1*. None of the cattle were found positive for trypanosome. We encountered only a few cattle in the field, and they were well kept and protected in an intensive management system, with proper care to prevent the spread of disease among them, whereas almost half the number of the pigs screened harbored trypanosome DNA (Table 3). Of the 65 pigs, 46.2% (95% CI = 34.6–58.1%) were positive for different *Trypanosoma* species. The predominant *Trypanosoma* species observed in pigs were *T. congolense* (forest type) and *T. simiae*. The trypanosome infection rate was greatest, at 100% each, at Nkatekwan, Santramor, and Zorh. There was a significant difference in trypanosome prevalence among communities (χ^2^ = 31.7, 95% CI = 34.6–58.1, *P* = 0.0002). The pigs were grouped into three age categories: <1 year, 1–2 years, and >2 years (Figure 5A). We encountered only two pigs that were older than 2 years, and they were both positive for a trypanosome parasite. Among pigs aged between 1 and 2 years, 29.2% (95% CI = 19.6–41.1%) were found to host trypanosomes. In addition, 12.3% of the young pigs (i.e., <1 year old; 95% CI = 6.4–22.5%) tested positive for the parasite out of a total of 33 examined. The study conducted on female and male pigs found no significant difference between the sexes in terms of the distribution of trypanosomes (Figure 5B).

**Table 3:** Distribution of trypanosome DNA within communities in the study area

Sampling Locations	Total Examined (*n*)	PCR Positive, *n* (%)	Odds Ratio (95% CI)	*P*-Value
Tsetse				
Adenta	71	45 (63.4)	Reference	Reference
Magazine	10	4 (40.0)	2.6 (0.7–8.7)	0.18
Nkatenkwan	49	31 (63.3)	1.0 (0.5–2.1)	0.99
Santramor	57	32 (56.1)	1.4 (0.7–2.8)	0.47
Teacher Mante	5	3 (60.0)	1.2 (0.2–5.9)	0.99
Whanabenya	4	2 (50.0)	1.7 (0.3–11.5)	0.63
Pinto	19	13 (68.5)	0.8 (0.3–2.5)	0.79
Zorh	6	5 (83.3)	0.3 (0.0–2.9)	0.42
Ayekokoso	8	0 (00.0)	∞ (3.3–∞)	0.00*
Pigs				
Magazine	7	1 (14.3)	Reference	Reference
Nkatenkwan	10	10 (100.0)	0.0 (0.0–0.2)	0.00*
Santramor	4	4 (100.0)	0.0 (0.0–0.4)	0.02
Teacher Mante	7	2 (28.6)	0.4 (0.0–4.7)	0.99
Whanabenya	7	1 (14.3)	1.0 (0.0–21.8)	0.99
Zorh	5	3 (60.0)	0.1 (0.0–1.4)	0.22
Bonkua No.1	7	2 (28.6)	0.4 (0.0–4.7)	0.99
Budu	7	1 (14.3)	1.0 (0.0–21.8)	0.99
Nankese	2	2 (100.0)	0.0 (0.0–0.9)	0.08
Mensakrom	7	2 (28.6)	0.4 (0.0–4.7)	0.99
Cattle				
Ayekokooso	10	0 (00.0)	Reference	Reference
Akorabo	10	0 (00.0)	4.3^303^ (0.0–5.6^309^)	0.99

PCR = polymerase chain reaction. The CIs (0.0–5.6) suggest that with 95% confidence, the actual odds ratio could be as low as 0.0 or as high as 5.6. The superscripts “303 and 309” in the odds ratio convey both the strength of the association and the level of certainty in the observed difference in trypanosome parasite distribution between the two communities.

* *P* <0.05 and highly significant.

### Molecular identification of tsetse blood meal source.

To identify the source of the blood meal, *cytochrome b* gene DNA was amplified from 10 tsetse that had taken blood meals. The samples were purified and prepared for sequencing. Five of the 10 samples gave readable DNA sequences, whereas the other five failed to produce readable sequences. The BLAST of the five DNA sequences identified the blood meal sources as one common warthog and four pigs (Table 4).

**Table 4:** Blood meal source determined by comparison with sequences of species in the National Center for Biotechnology Information GenBank database

Mammalian Host Species	*Cytochrome b* Detection Rate	GenBank Accession No.
*Phacochoerus africanus* (common warthog)	1/28*n/N*	KJ192881
*Sus scrofa* breed, Fengjing mitochondrion (pig)	4/28*n/N*	MH603005.1

*n* = sub-samples; *N* = total samples examined.

## DISCUSSION

This study identified the presence of *G*. *palpalis* in the forest zone of the eastern region and corroborates the report by other studies that investigated the distribution of tsetse in the same region.^17^^,^^18^ Tsetse species have varying ecological requirements to thrive in a particular environment.^37^ For instance, *G. palpalis* prefers the forest, in that desired environmental factors are invariable.^38^ However, other species such as *Glossina morsitans* and *Glossina tachinoides*, which have been reported previously in northern Ghana,^39^ were absent in our study. According to the tsetse distribution models, *G. morsitans* belonging to the subgenus *Nemorhina* typically inhabits areas near water sources such as riverine forests and vegetation along lakes.^40^ Thus, the absence of several species, including *G. morsitans* and *G. tachinoides*, as reported herein and in other studies,^41^ could be an indication of a predisposition toward a northerly retreat of these species. All tsetse species can transmit trypanosomes; however, the *G. palpalis* group is known to be the most significant transmitting vector for trypanosomiasis.^42^ It is capable of transmitting *Trypanosoma brucei gambiense*, which causes sleeping sickness in both western and central Africa.^43^ It is also efficient in transmitting animal pathogenic trypanosomes, including *T. vivax* and *T. congolense.*

Apparent densities did not differ significantly among the tsetse biotopes. The overall apparent density recorded was 4.3 tsetse per trap per day. However, when compared with previous studies^44^^,^^45^ conducted in other parts of the Saharan subregion, the apparent density in our study is less. This may be a result of the different geographic locations as well as the vegetative cover.

### Trypanosome DNA in tsetse.

More than half of the investigated tsetse were found to be carrying trypanosome DNA, and this makes the occurrence rate high compared with that of a previous study^46^ in the same region. However, the rate was less than the prevalence recorded in a recent study^47^ conducted in the northern part of the country. Overall trypanosome positivity in the tsetse gut was greater than that in the proboscis in our research, which agrees with previous studies^48^^,^^49^ conducted in other parts of West Africa. There was evidence of multiple infections of trypanosomes in the tsetse gut^50^ but not in the proboscis. In most studies, trypanosome occurrence is always high in the tsetse gut compared with the mouthparts.^51^ This is because whenever a tsetse takes up an infected blood meal, several trypanosome parasites are mostly cleared away by the tsetse immune system.^52^ For this reason, many trypanosomes are unable to mature and migrate from the gut to the salivary gland and the mouthparts.^53^ Therefore, the trypanosome DNA rates in the salivary gland and the proboscis are always low compared with that in the gut.^54^ It is good to note that DNA is relatively stable and can circulate in body tissues over a while, and PCR is a relatively sensitive molecular tool to detect trypanosome DNA in varying hosts at varying times.^55^ So the detection of DNA may be enough to determine an active infection. Therefore, the percentages shown here may reflect an ongoing transmission that needs to be monitored.

As mentioned earlier, more female tsetse collected in our study were positive for trypanosome DNA than were male tsetse. The lower occurrence of trypanosomes in male tsetse compared with female tsetse could be attributed to the imbalanced ratio between male and female tsetse populations. In another research finding, male tsetse exhibited higher susceptibility than female tsetse.^56^ A greater proportion of female tsetse were found to carry trypanosome DNA compared with male tsetse. That potentially influenced the imbalanced sex ratios of tsetse samples collected during the study, leading to a statistical bias toward females. *Trypanosoma congolense* was the dominant *Trypanosoma* species in the study area, with a prevalence of 45.8%. This was higher than that reported in a study carried out in the same region.^46^ Ngomtcho et al.^48^ found a much lower infection rate (24.8%) of *T. congolense* in the same vector species, *G. palpalis*, in northern Cameroon. There was evidence of concurrent colonization of mixed trypanosome infection in the tsetse gut (Figure 5C). This might come from tsetse blood meals infected with different *Trypanosoma* species or successive blood meal uptake from different pigs with different *Trypanosoma* species.

In our study, five of the sampling sites were situated on cocoa farms surrounded by dense secondary forest, whereas the remaining sites were mostly shrubs with few trees sparsely distributed. Our findings indicate that trypanosome parasites were present at all locations where pigs were found, except for Ayekokooso and Akorabo, where the two cattle ranches were also situated. We also found that sites close to dense vegetation had more tsetse and frequent trypanosome occurrences than sites with relatively less dense vegetation. There were statistically significant differences among the different sites (χ^2^ = 31.7, *P* <0.05), highlighting the importance of dense vegetation and the presence of pigs as significant risk factors for the transmission of AAT. This pattern was consistent with that observed in a previous study conducted in Kiwanga village, which is located close to the Kiwanga forest.^57^

### *Trypanosoma* species in pigs and cattle.

Our study revealed trypanosome infection rates of 46.1% and 0% in pigs and cattle, respectively. We found only two cattle ranches in the study area, and both housed 10 cattle each. We sampled all 20 cattle, which belonged to different breeds (e.g., Zebu, Ndama, Sanga, Guda), but none of the samples were positive for trypanosome parasites. We observed that the cattle were maintained in an intensive management system, in which frequent access to veterinary services for the cattle was a routine farm practice. Ranchers also affirmed that the cattle were being sprayed occasionally to prevent tsetse and other biting. So, it is not surprising that the cattle were not positive for trypanosome DNA. However, we will not make any conclusive statements; instead, we present the findings as they are. Nevertheless, additional research is required to gain a more comprehensive understanding of the occurrence and prevalence of these parasites in livestock.

Concerning the pigs, the infection rate was greater than that reported in a study carried out in the same region,^17^ and the results were also consistent with studies conducted in other parts of Africa.^58^^–^^60^ This may suggest that pigs are major reservoirs of trypanosomiasis, as reported in other studies.^61^ This also implies that other domesticated animals such as dogs and goats in the area need to be investigated because they may be harboring parasites reported in other parts of Africa.^59^^,^^62^^,^^63^ The greater trypanosome occurrence may be attributed in part to poor pig production management. We observed that pig production in rural communities was mostly semi-intensive, with neither veterinary services nor any interventional measures to prevent tsetse–pig contact. Although a few farms were found to have tattered nets around pigsties, they were not enough to prevent the pigs from tsetse bites. Evidence for this is the high trypanosome DNA positivity rate recorded in most of the pigs examined in our study. Three *Trypanosoma* species were identified in the examined pigs, with *T. congolense* being the dominant species. This corroborates the findings of Dale et al.^64^ and others,^65^ in which ∼20% and 16% of pigs tested carried *T. simiae* and *T. vivax,* respectively. The occurrence of *T. vivax* in pigs, as found in our study, is consistent with the results found in a study by Penchenier et al.^66^ in which species-specific PCR was performed to reveal high levels of *T. vivax* infection in pigs. Interestingly, we observed that high trypanosome prevalence in tsetse corresponded to an increase in trypanosome DNA in pigs from the same sampling site. In our study, we sampled pigs of different age groups and found that the risk of trypanosome occurrence increased with age, as χ^2^ analysis revealed a significant difference among the age groups (*P* <0.05). This could be a result of the fact that older pigs may have had longer exposure to tsetse bites and that piglets may not have had any tsetse contact before sampling was conducted. However, further studies with larger sample sizes are needed to draw more conclusive results.

### Blood meal analysis.

The availability of a blood meal source is one of the most important factors for tsetse to thrive in a habitat.^67^ Thus, the presence of vertebrate hosts, including pigs, is a key indicator of the presence of the tsetse fly in an area. Nevertheless, tsetse are known to obtain a blood meal from a wide range of vertebrate hosts^68^ and sometimes adapt to feeding on a new host in the absence of the usual host.^69^ Sequence analysis of the *ctb* gene of five tsetse yielded greater fragment resolution, which allowed for easy identification of two vertebrates—*Sus scrofa domesticus* (domestic pig) and *Phacochoerus africanus* (warthog)—as host species of the few tsetse that were blood fed (Table 4). The tsetse were collected at Santramor, a community surrounded by secondary forests that could serve as a habitat for both the tsetse and the wild animals. This was consistent with studies carried out by Ebhodaghe et al.^70^ in Kenya. The other five samples gave low-quality, unreadable fragments and could not be resolved. This failure could be a result of DNA damage, which can cause significant errors in DNA sequencing.^71^ Such fragments could represent interesting species, and further studies are required to identify the host preference of the tsetse and appropriate tsetse control measures.

### Limitations of the study.

We could not investigate the salivary gland because of a lack of expertise in salivary gland dissection. The salivary gland, if investigated, could have provided information on more mature trypanosomes. Moreover, a limited number of livestock production farms were observed in the study area. We came across just two cattle ranches, housing a combined total of 20 cattle. A greater number of cattle could have produced a more interesting result than what we obtained. However, we sought to monitor the presence of trypanosome parasites circulating in cattle and pigs in the study area, not necessarily the number of samples encountered. Again, the number of biconical traps used in our study was few but was directly proportional to the number of pigsties and cattle ranches we encountered in the field.

## CONCLUSION

The outcome of our study revealed that *G. palpalis* is the only tsetse species (see the phylogenetic tree in Supplemental Figure 1) in the Suhum/Kraboa/Coaltar districts responsible for the transmission of trypanosomes in pigs. The presence of the *G. palpalis* group in the area may pose a public health challenge because a high percentage of the tsetse carried the DNA of trypanosome parasites. The occurrence level of parasites in the examined pigs elucidates the risk involved in the transmission of the parasite in livestock and possibly people in the area, who may be exposed to the bite of the tsetse. Also, the rate of trypanosome parasites in tsetse and pigs indicates that animal–tsetse control measures may be lacking in the study area. It is recommended that further studies involving a wide range of domestic animals be carried out to determine the prevalence and risk factors of nagana in different seasons and establish seasonal variation in animal trypanosomiasis transmission. Active surveillance is essential to monitor the transmission cycle of AAT because livestock serve as reservoirs for human African trypanosomiasis.

## Supplemental Materials

10.4269/ajtmh.23-0329 Supplemental Materials

## Data Availability

The datasets generated and/or analyzed during our study are available in Harvard Dataverse Replication Data for the transmission of African animal trypanosomiasis (nagana) in two districts in the forest zone of Ghana, available at https://doi.org/10.7910/DVN/IELHN5.
