# Supplementary material for: The Transmission of Animal African Trypanosomiasis in Two Districts in the Forest Zone of Ghana
Source: Am J Trop Med Hyg. 2024 May 2;110(6):1127–36. doi: 10.4269/ajtmh.23-0329 (PMC11154048; doi:10.4269/ajtmh.23-0329)
Supplement: Supplemental Materials [file tpmd230329.SD1.pdf]

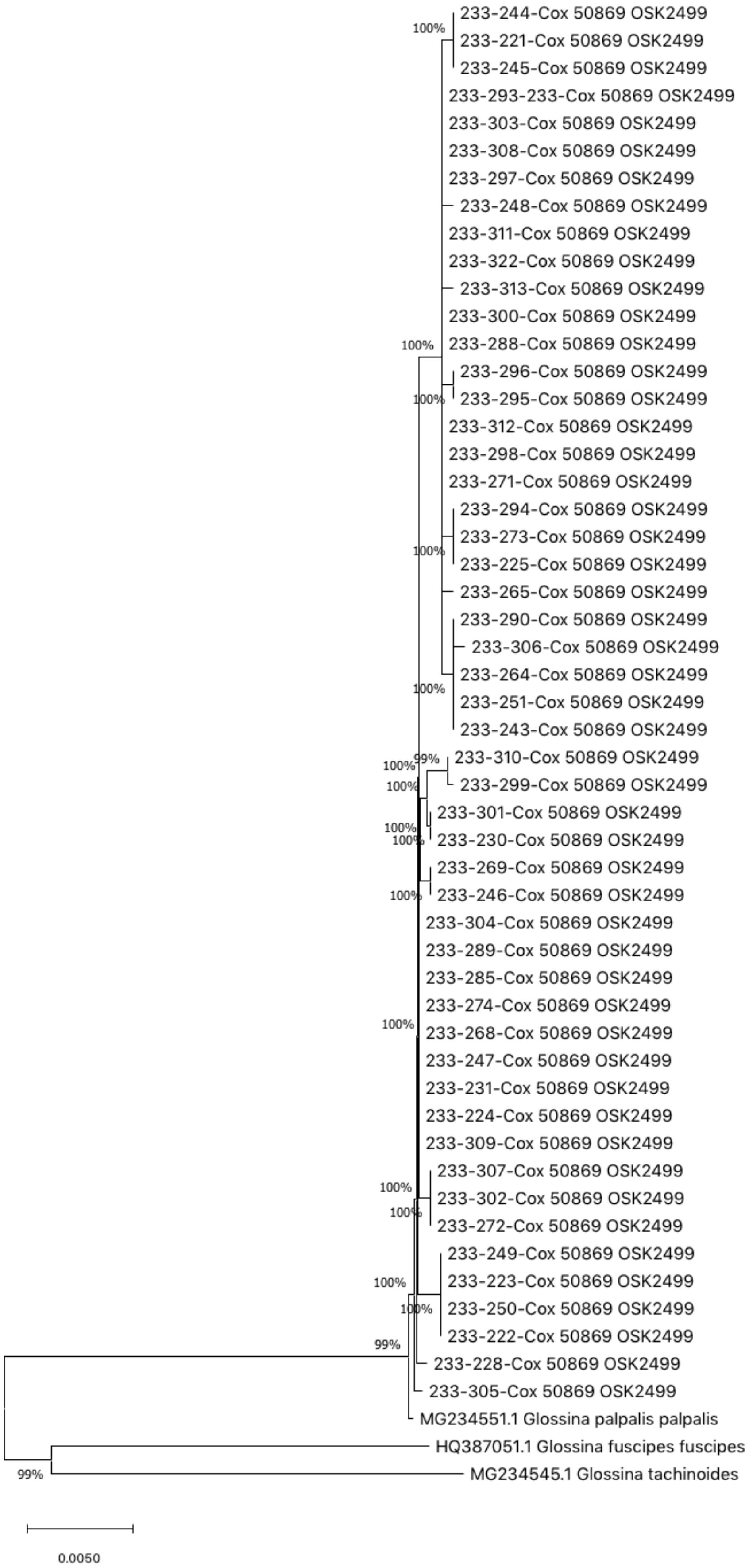
233-244-Cox 50869 OSK2499
233-221-Cox 50869 OSK2499
233-245-Cox 50869 OSK2499
233-293-233-Cox 50869 OSK2499
233-303-Cox 50869 OSK2499
233-308-Cox 50869 OSK2499
233-297-Cox 50869 OSK2499
233-248-Cox 50869 OSK2499
233-311-Cox 50869 OSK2499
233-322-Cox 50869 OSK2499
233-313-Cox 50869 OSK2499
233-300-Cox 50869 OSK2499
233-288-Cox 50869 OSK2499
233-296-Cox 50869 OSK2499
233-295-Cox 50869 OSK2499
233-312-Cox 50869 OSK2499
233-298-Cox 50869 OSK2499
233-271-Cox 50869 OSK2499
233-294-Cox 50869 OSK2499
233-273-Cox 50869 OSK2499
233-225-Cox 50869 OSK2499
233-265-Cox 50869 OSK2499
233-290-Cox 50869 OSK2499
233-306-Cox 50869 OSK2499
233-264-Cox 50869 OSK2499
233-251-Cox 50869 OSK2499
233-243-Cox 50869 OSK2499
233-310-Cox 50869 OSK2499
233-299-Cox 50869 OSK2499
233-301-Cox 50869 OSK2499
233-230-Cox 50869 OSK2499
233-269-Cox 50869 OSK2499
233-246-Cox 50869 OSK2499
233-304-Cox 50869 OSK2499
233-289-Cox 50869 OSK2499
233-285-Cox 50869 OSK2499
233-274-Cox 50869 OSK2499
233-268-Cox 50869 OSK2499
233-247-Cox 50869 OSK2499
233-231-Cox 50869 OSK2499
233-224-Cox 50869 OSK2499
233-309-Cox 50869 OSK2499
233-307-Cox 50869 OSK2499
233-302-Cox 50869 OSK2499
233-272-Cox 50869 OSK2499
233-249-Cox 50869 OSK2499
233-223-Cox 50869 OSK2499
233-250-Cox 50869 OSK2499
233-222-Cox 50869 OSK2499
233-228-Cox 50869 OSK2499
233-305-Cox 50869 OSK2499
MG234551.1 Glossina palpalis palpalis
HQ387051.1 Glossina fuscipes fuscipes
MG234545.1 Glossina tachinoides
100%
99%
0.0050

Evolutionary relationships of taxa

The evolutionary history was inferred using the Neighbor-Joining method [1]. The optimal tree with the sum of branch length = 0.06951146 is shown. The percentage of replicate trees in which the associated taxa clustered together in the bootstrap test (700 replicates) are shown next to the branches [2]. The tree is drawn to scale, with branch lengths in the same units as those of the evolutionary distances used to infer the phylogenetic tree. The evolutionary distances were computed using the Maximum Composite Likelihood method [3] and are in the units of the number of base substitutions per site. The proportion of sites where at least 1 unambiguous base is present in at least 1 sequence for each descendent clade is shown next to each internal node in the tree. This analysis involved 54 nucleotide sequences. Codon positions included were 1st+2nd+3rd+Noncoding. All ambiguous positions were removed for each sequence pair (pairwise deletion option). There was a total of 762 positions in the final dataset. Evolutionary analyses were conducted in MEGA X [4].

1. Saitou N. and Nei M. (**1987**). The neighbor-joining method: A new method for reconstructing phylogenetic trees. *Molecular Biology and Evolution* **4**:406-425.
2. Felsenstein J. (**1985**). Confidence limits on phylogenies: An approach using the bootstrap. *Evolution* **39**:783-791.
3. Tamura K., Nei M., and Kumar S. (**2004**). Prospects for inferring very large phylogenies by using the neighbor-joining method. *Proceedings of the National Academy of Sciences* (*USA*) **101**:11030-11035.
4. Kumar S., Stecher G., Li M., Knyaz C., and Tamura K. (**2018**). MEGA X: Molecular Evolutionary Genetics Analysis across computing platforms. *Molecular Biology and Evolution* **35**:1547-1549.

Disclaimer: Although utmost care has been taken to ensure the correctness of the caption, the caption text is provided "as is" without any warranty of any kind. Authors advise the user to carefully check the caption prior to its use for any purpose and report any errors or problems to the authors immediately (www.megasoftware.net). In no event shall the authors and their employers be liable for any damages, including but not limited to special, consequential, or other damages. Authors specifically disclaim all other warranties expressed or implied, including but not limited to the determination of the suitability of this caption text for a specific purpose, use, or application.
